# Development of Fluorescence-Based Assays for Key Viral Proteins in the SARS-CoV-2 Infection Process and Lifecycle

**DOI:** 10.3390/ijms25052850

**Published:** 2024-03-01

**Authors:** Mingzhenlong Deng, Chuang Zhang, Wanli Yan, Lei Chen, Bin He, Yan Li

**Affiliations:** 1 State Key Laboratory of Functions and Applications of Medicinal Plants, Engineering Research Center for the Development and Application of Ethnic Medicine and TCM (Ministry of Education), Guizhou Provincial Key Laboratory of Pharmaceutics, School of Pharmacy, Guizhou Medical University, Guiyang 550004, China; dengmingzhenlong@163.com (M.D.); zc13116478432@163.com (C.Z.); y2577532533@163.com (W.Y.); leichen@gmc.edu.cn (L.C.); 2 School of Basic Medical Science, Guizhou Medical University, Guiyang 550004, China

**Keywords:** COVID-19, SARS-CoV-2, infection, assay development, antiviral agents

## Abstract

Since the appearance of SARS-CoV-2 in 2019, the ensuing COVID-19 (Corona Virus Disease 2019) pandemic has posed a significant threat to the global public health system, human health, life, and economic well-being. Researchers worldwide have devoted considerable efforts to curb its spread and development. The latest studies have identified five viral proteins, spike protein (Spike), viral main protease (3CLpro), papain-like protease (PLpro), RNA-dependent RNA polymerase (RdRp), and viral helicase (Helicase), which play crucial roles in the invasion of SARS-CoV-2 into the human body and its lifecycle. The development of novel anti-SARS-CoV-2 drugs targeting these five viral proteins holds immense promise. Therefore, the development of efficient, high-throughput screening methodologies specifically designed for these viral proteins is of utmost importance. Currently, a plethora of screening techniques exists, with fluorescence-based assays emerging as predominant contenders. In this review, we elucidate the foundational principles and methodologies underpinning fluorescence-based screening approaches directed at these pivotal viral targets, hoping to guide researchers in the judicious selection and refinement of screening strategies, thereby facilitating the discovery and development of lead compounds for anti-SARS-CoV-2 pharmaceuticals.

## 1. Introduction

Coronavirus belongs to the enveloped, single-stranded, positive-stranded RNA virus family [1] that is widely spread between humans and animals and can cause mild to severe respiratory infections in humans as well as intestinal, liver and nervous system diseases. At the beginning of the 21st century, the coronavirus triggered two large-scale epidemics around the world, including the severe acute respiratory syndrome (SARS) in 2002 and the Middle East respiratory syndrome (MERS) in 2012 [2,3], which caused huge population casualties and economic losses [4,5]. 

In December 2019, a new type of coronavirus (2019-nCoV) appeared in Wuhan, Hubei Province, China [6]. This novel coronavirus has exhibited extremely high infectivity and spread rapidly on a global scale. From 2019 to the present, it has far surpassed SARS and MERS in terms of the number of infections, deaths, and the spatial extent of the epidemic [7], leading to an unprecedented outbreak of viral pneumonia worldwide, posing a huge threat to human health and the global health system [8].

The rapid spread of the SARS-CoV-2 virus is associated with its reliance on airborne transmission of respiratory droplets and aerosols, close contact between infected individuals, as well as the infectivity of asymptomatic carriers [9]. Patients with COVID-19 may have severe hypoxemia, viral pneumonia, acute respiratory distress syndrome, and gastrointestinal and neurological symptoms [10].

The strong infectious ability of the novel coronavirus and the serious complications caused by it have quickly attracted the attention of countries and related organizations around the world. On 30 January 2020, the World Health Organization (WHO) announced that the novel coronavirus epidemic was listed as a public health emergency of international concern [11]. On 11 February 2020, the International Commission on Taxonomy of Viruses named the novel coronavirus “SARS-CoV-2” [12], while the WHO named the disease caused by the novel coronavirus “COVID-19” and announced the possibility of a worldwide pandemic of novel coronavirus pneumonia on 11 March 2020 [13].

In order to identify potential targets from the virus itself that can inhibit its spread, replication, and proliferation, and to curb the continued spread of the epidemic worldwide, researchers have used sub-atomic resolution three-dimensional averaging (STA) and cryo-electron tomography (Cryo-ET) techniques to reveal the true molecular structure of SARS-CoV-2 [14]. Additionally, the genome of SARS-CoV-2 has been analyzed and sequenced, revealing that it comprises 14 open reading frames (ORFs), two-thirds of which are responsible for encoding 16 nonstructural proteins (NSP1-16), forming a replication enzyme complex. The remaining one-third encodes nine accessory proteins and four structural proteins: spike protein (S), envelope protein (E), membrane protein (M), and nucleocapsid protein (N) [15]. These proteins have high sequence similarity to the corresponding proteins of SARS-CoV and MERS-CoV, and the genome sequence of SARS-CoV-2 is very similar to that of SARS-CoV and MERS-CoV [16]. This may indicate that the strategies for the treatment of SARS-CoV and MERS-CoV are also applicable to SARS-CoV-2, and effective anti-SARS-CoV-2 drugs can also be developed based on the same targets.

To date, small-molecule antiviral drugs (such as nirmatrelvir–ritonavir, remdesivir, molnupiravir, dexamethasone and baricitinib) and monoclonal antibodies (such as bebtelovimab, sotrovimab and regdanvimab) have been approved for the treatment of COVID-19 [17]. However, there are some problems, such as the new use of old drugs, the toxic and side effects of drugs, the poor pharmacokinetic properties and the poor therapeutic effect on mutant viruses [18]. So far, no drugs have been shown to be generally effective against different severities of infection and mutant virus strains, especially for patients with low immunity and severe infection of SARS-CoV-2 [19]. Therefore, it is urgent to find new therapeutic targets to develop anti-SARS-CoV-2 drugs, which are not difficult to find from the currently approved anti-SARS-CoV-2 drugs in the world (Table 1). Drugs (Sotrovimab, Regdanvimab, Bamlanivimab/Etesevimab, Tixagevimab/Cilgavimab, Bebtelovimab, Remdesivir, Molnupiravir, Favipiravir, Azvudine, VV116, Nirmatrelvir/Ritonavir, Ensitrelvir) developed for several important proteins in SARS-CoV-2 infection and life cycle have been approved for marketing, indicating that developing more anti-SARS-CoV-2 drugs for these viral proteins is very promising.

## 2. Promising Therapeutic Targets for the Novel Coronavirus (SARS-CoV-2)

SARS-CoV-2 is an enveloped, positive-strand, single-stranded RNA virus of the genus β-coronavirus [20]. Like all coronaviruses, SARS-CoV-2 contains a spike glycoprotein(S) on the surface. The S protein is indispensable for viral replication because it mediates the entry of the virus into the cell and is highly correlated with the virus’s ability to infect [21,22]. The S protein contains two functional subunits: S1 and S2. S1 is responsible for recognizing the receptor-angiotensin-converting enzyme2 (ACE2) on the surface of the host respiratory cell through its C-terminal receptor binding region (RBD) to enter the cell and promote viral infection. S2 contains the basic elements required for the membrane fusion process, mainly mediating the fusion of the virus and host cell membrane [10,21]. The entry of SARS-CoV-2 into cells is largely dependent on the hydrolysis of TMPRSS 2 protein to activate the S protein on the surface of the virus in addition to a TMPRSS 2-independent route, and the virus–cell membrane fusion is carried out to mediate the entry of the virus into the cell. [23]. After SARS-CoV-2 enters the host cell, the released and uncoated large segment (>30 kb) viral RNA genome will produce two open reading frames: ORF1a and ORF1b. The first round of translation of these two ORFs produces PP1A and PP1B polyproteins, which are subsequently cleaved by papain-like protease (PLpro) and 3C protease-like protease (3CLpro) to produce nonstructural proteins (NSPs), such as RNA-dependent RNA polymerase (RdRp) and helicase [24,25,26]. RDRP can be used to complete the transcription and synthesis of negative-strand subgenomic RNA, the synthesis of mRNAs related to different structural proteins, and the replication of viral genomic RNA [25]. Helicase plays a key role in the unwinding of positive and negative-strand viral RNA and the replication of a large number of viral RNA [27]. 

**Figure 1:**
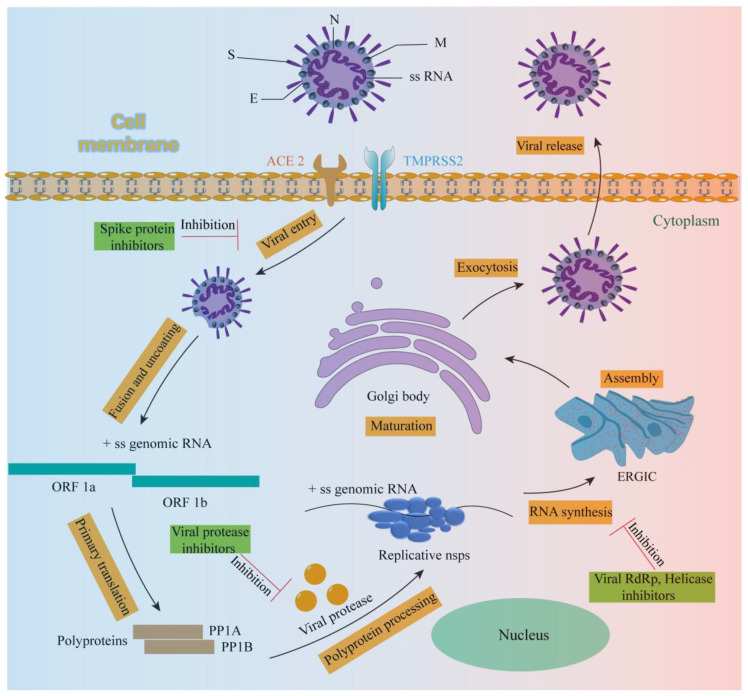
Virus body and life cycle of SARS-CoV-2.

Therefore, five viral protein targets, Spike, 3CLpro, PLpro, RdRp and Helicase (Table 2), play a key role in the entire replication cycle of SARS-CoV-2 (Figure 1). As to these five viral targets, there is great hope to develop effective treatment strategies that would (1) interfere with the binding of viral Spike protein to the ACE2 receptor on the surface of host cells to prevent viral infection of cells [22,28], (2) inhibit the activity of PLpro and 3CLpro, interfering with their cleavage of multiple proteins to produce nonstructural proteins (NSPs) such as RdRp and helicase [29], (3) antagonize RdRp activity and block viral RNA transcription, synthesis and replication [25,30], and (4) interfere with the role of viral helicase and inhibit the replication of viral RNA [27]. Relying on these five viral protein targets to establish efficient drug screening methods may be convenient for finding natural products with anti-SARS-CoV-2 potential [31,32]. 

At present, computer simulation screening methods based on ligand-based machine learning and molecular docking [33,34], screening methods based on cytopathic effect (CPE) [35], and fluorescence-based drug screening methods have been developed for these targets. While the fluorescence-based method has inherent drawbacks, such as sensitivity to background fluorescence interference and quenching effects, limited selectivity across all compounds, and a narrow applicability range, it remains a favored approach in drug lead compound screening. Despite its limitations, the fluorescence-based screening method stands out in the drug discovery process due to its rapidity, efficiency, and clarity of results. Researchers have addressed its shortcomings through optimization and adaptation to unique targets. Consequently, the fluorescence screening method holds great promise for effectively screening drugs with potential anti-SARS-CoV-2 activity. 

Recently, many applications of fluorescence technology in the diagnosis of SARS-CoV-2 and the development of anti-SARS-CoV-2 drugs have been published, which proves that fluorescence technology has great application prospects in the field of anti-SARS-CoV-2 [36,37]. Many fluorescence screening methods targeting key viral proteins of SARS-CoV-2 have been developed, and these methods have been used to screen a large number of compounds with anti-SARS-CoV-2 activity, providing numerous novel and promising lead compounds for the development of new SARS-CoV-2 drugs [38,39]. Therefore, we review the current fluorescence screening methods developed for these five viral targets, hoping to provide help for relevant researchers in method selection.

## 3. Fluorescence-Based Drug Screening Method for Novel Coronavirus (SARS-CoV-2)

### 3.1. Fluorescence-Based Drug Screening Method Targeting Spike Protein

The entry of SARS-CoV-2 into cells depends on two key procedures: the activation of the viral surface S protein by TMPRSS 2 protein hydrolysis, and the binding of the activated S protein (S1) to the cell surface receptor ACE2 for virus–cell membrane fusion [40,41]. Therefore, interfering with the binding of S protein to ACE2 will prevent the virus from invading cells. Many compounds may have the potential activity of inhibiting the binding of S protein to ACE2, but we need to screen and hit them from a large number of compound libraries, so it is necessary to establish an efficient and rapid screening method. At present, many fluorescence-based high-throughput screening methods have been developed to screen compounds that can interfere with the binding of the S protein to its host cell receptor ACE2.

**Figure 2:**
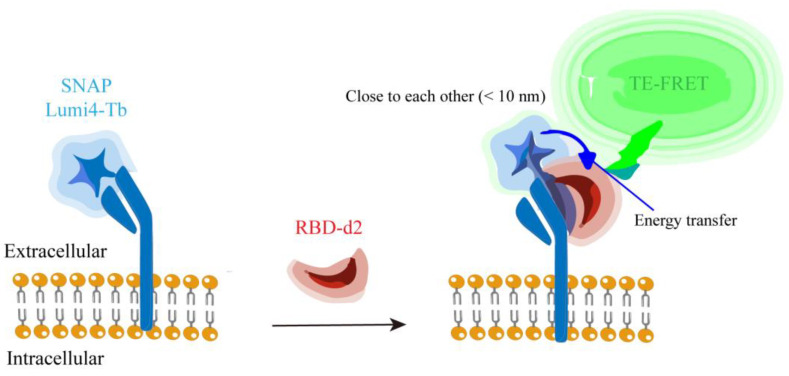
The scheme elucidates the binding assay between RBD-d2 and SNAP-tagged ACE2 labeled with Lumi4-Tb based on TR-FRET.

Time-resolved fluorescence resonance energy transfer (TR-FRET) is a fluorescence-based technique. The principle of TR-FRET is based on time-resolved fluorescence (TRF) measurement and fluorescence resonance energy transfer (FRET) between donor and acceptor molecules. This technique can analyze molecular interactions in biochemical processes. TR-FRET technology is widely used to study kinase assay, cell signal transduction pathway, protein–protein interaction, DNA–protein interaction and receptor–ligand binding [42]. Many researchers have developed it as a screening method for SARS-CoV-2 virus protein and enzyme activity. Erika Cecon et al. devised a high-throughput screening technique utilizing the principle of TR-FRET. As shown in Figure 2, SNAP is an O6-alkylguanine-DNA alkyltransferase that is capable of catalyzing its own covalent binding to fluorescent derivatives of benzylguanine, such as Lumi4-Tb. This determination is based on energy transfer between energy donors (terbium [Tb]-labeled N-terminal SNAP-labeled human ACE2, SNAP-ACE2) and energy receptors (d2 fluorophore-labeled SARS-CoV-2 spike protein RBD, RBD-d2). This energy transfer occurs only when the two are close to each other (<10 nm), and then fluorescence is generated [43]. Therefore, when there are compounds that potentially inhibit the binding of S protein and ACE2, the fluorescence generated by the combination of the two (energy donor and energy receptor are close) will be affected. Thus, the target compounds can be effectively screened [22].

**Figure 3:**
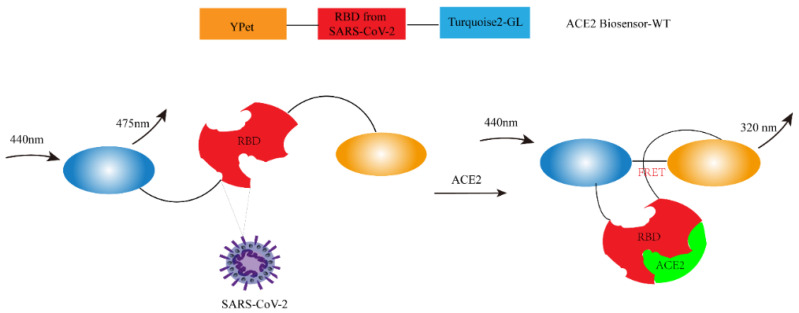
Design and characterization of the FRET-based ACE2 biosensor.

Fluorescence resonance energy transfer (FRET) is a nonradiative process of energy transfer. Its principle is based on the dipole–dipole interaction between fluorescent molecules. The transfer of energy from donor molecules to side-by-side molecules (such as 0 to 10 nm) occurs rapidly and produces fluorescence. FRET occupies a central position in biotechnology and biological research. The pharmaceutical industry has developed many FRET-based fluorescence screening methods [44], including fluorescence screening methods for SARS-CoV-2. Jung-Soo Suh et al. developed a novel ACE2 biosensor based on the RBD module derived from SARS-CoV-2 using FRET technology. This biosensor demonstrates high spatial and temporal resolution and effectively monitors the activity of ACE2. As depicted in Figure 3, the ACE2 biosensor is composed of a YPet energy receptor, a SARS-CoV-2-derived RBD domain, and a truncated Turquoise 2-GL energy donor. Upon binding to ACE2, the ACE2 biosensor undergoes significant conformational changes, resulting in an increase in the FRET/CFP ratio [45]. The biosensor enables efficient and rapid screening of lead compounds that inhibit the interaction between hACE2-RBD and SARS-CoV-2. It can be safely used for studying potential drugs against SARS-CoV-2 without the necessity of directly handling the virus.

**Figure 4:**
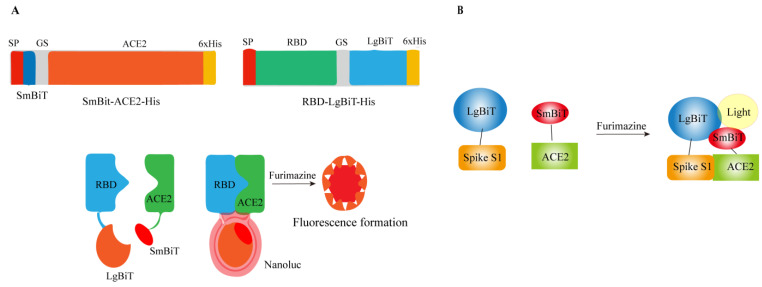
Construction of RBD: ACE 2 NanoBiT biosensor. (**A**) The structure diagram of SRAE 2-BS and the demonstration of its working mechanism. (**B**) Schematic diagram of Nano-luciferase complementation-based biosensor for the interaction between SARS-CoV-2 Spike S1 protein and ACE2 ectodomain.

In 2016, an ultrasensitive lysed luciferase complementation assay (SLCA) called NanoLuc binary technology (NanoBiT) was developed to monitor protein–protein interaction PPIs. The determination is based on the following: NanoLuc luciferase can be divided into two fragments, namely an 18-kDa large BiT (LgBiT or Lg) and an 11-amino acid small BiT (SmBiT or Sm). Each of these fragments is fused with the target protein. When the target proteins interact, it leads to the reconstitution of LgBiT and SmBiT, resulting in the activation of NanoLuc luciferase. In the presence of its substrate furimazine, this activated luciferase emits light [46]. Based on this principle, Xiaolong Yang et al. developed the first NanoBiT biosensor to quantitatively measure the protein–protein interaction (PPI) between SARS-CoV-2 RBD and ACE2 both in vitro and in vivo. The biosensor plasmid was created by fusing either LgBiT or SmBiT with RBD or ACE2 at either the N-terminus or C-terminus. When RBD and ACE2 interact with each other, LgBiT and SmBiT can recombine to form an active NanoLuc luciferase, which emits light in the presence of its substrate furimazine, as shown in Figure 4A [47]. 

Based on the same principle as Xiaolong Yang et al., Taha Azad et al. developed and optimized the Nano Bi T biosensor (Figure 4B). The large dynamic range, enhanced thermal stability and pH tolerance, and versatility of the biosensor were demonstrated [48]. This rapid in vitro screening tool does not necessitate the use of live viruses and is expected to expedite the development of effective antiviral strategies targeting the SARS-CoV-2 infected cell pathway.

**Figure 5:**
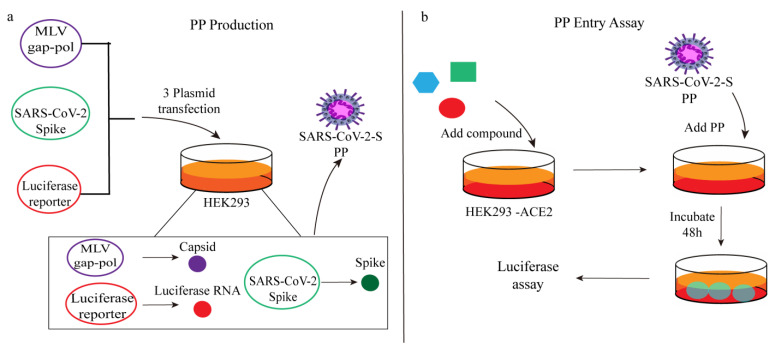
(**a**) PP production and (**b**) PP entry assay schematics.

Miao Xu et al. established a cell-based SARS-CoV-2 entry assay using viral pseudoviruses. Firstly, HEK 293 cells were transfected with three plasmids (including MLV capsid protein, SARS-CoV-2 spike protein and luciferase RNA) to produce pseudovirus particles (PP). The outer surface of the obtained PP carried SARS-CoV-2 spike protein, which was then added to HEK 293-ACE2 cells. SARS-CoV-2 spike protein specifically interacts with the ACE2 receptor on the cell surface, triggering a series of cascade reactions. This interaction prompts the host protease to initiate the endocytosis of pseudoviral particles and promotes the fusion between the particle envelope and the cell membrane. It is important to note that the pseudoparticles employed in this assay carry firefly luciferase RNA instead of the actual SARS-CoV-2 genome, rendering them incapable of replication within the cells. The entry process of the pseudoparticles is considered complete once membrane fusion occurs and the luciferase RNA is released into the cell. After an incubation period of 48 h, the expression of luciferase can be detected, generating a fluorescence signal (Figure 5) [49]. The amount of PP entering can be evaluated by measuring the fluorescence signal via the activation of luciferase.

**Figure 6:**
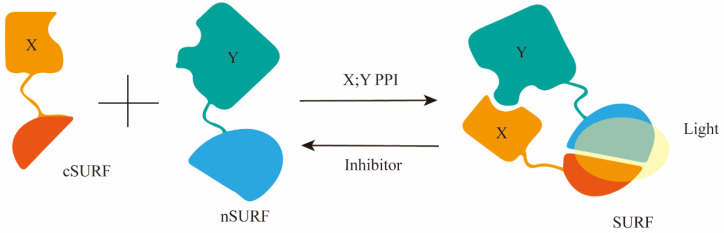
A diagram illustrating the structure-based design of the PPI reporter SURF, utilizing split fluorescent proteins, and the initial SURF reporter gene for imaging the interaction between S protein and ACE2 at its peak.

Junjiao Yang et al. improved upon the existing green fluorescent protein–protein interaction (PPI) reporter and developed a new fluorescent reporter called SURF (split UnaG-based reversible and fluorogenic PPI reporter) for detecting the interaction between the S protein and ACE2. Figure 6 shows that based on crystal structure analysis of the S protein-ACE2 complex, it was discovered that the PPI occurs through the receptor binding domain (SRBD) of the S protein. Therefore, they fused the C-terminal component of SURF (cSURF) to SRBD and the N-terminal component of SURF (nSURF) to ACE2. The interaction between SRBD and ACE2 brings the two SURF fragments close together, allowing them to recombine and emit fluorescence. As SURF is reversible, compounds that inhibit PPI cause the dissociation of the two SURF fragments, leading to loss of fluorescence [50]. Therefore, it can be utilized for screening compounds that inhibit the interaction between the S protein and ACE2.

**Figure 7:**
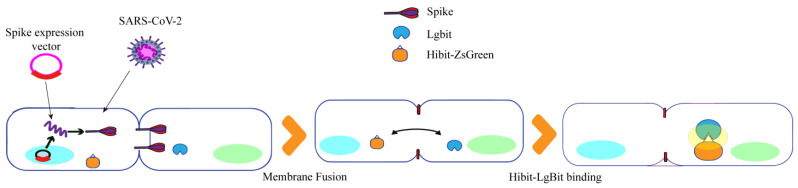
Luciferase quantitative detection of SARS-CoV-2S protein-induced membrane fusion design diagram.

Kei Haga et al. developed a quantitative detection method for SARS-CoV-2 S protein-induced membrane fusion using luciferase. Figure 7 illustrates their approach based on HiBit technology, which involves the binding of a small 11-amino acid peptide called HiBit to a larger subunit called LgBit with high affinity, forming a complex with luciferase activity. Firstly, HiBit was attached to the C-terminus of green fluorescent protein (ZS), and Vero/TMPRSS 2 cells expressing ZsGreen-HiBit or LgBit were co-cultured in the same well. Subsequently, Vero E6 cells expressing TMPRSS 2 (Vero/TMPRSS 2) were infected with SARS-CoV-2, resulting in significant cell fusion. The fused ZsGreen-HiBit and LgBit formed a highly active luciferase complex, allowing for quantification of the virus-induced membrane fusion by measuring the change in fluorescence signal value [51]. Therefore, the HiBit-LgBit system can be used to screen drugs against SARS-CoV-2, especially for virus entry and membrane fusion.

**Figure 8:**
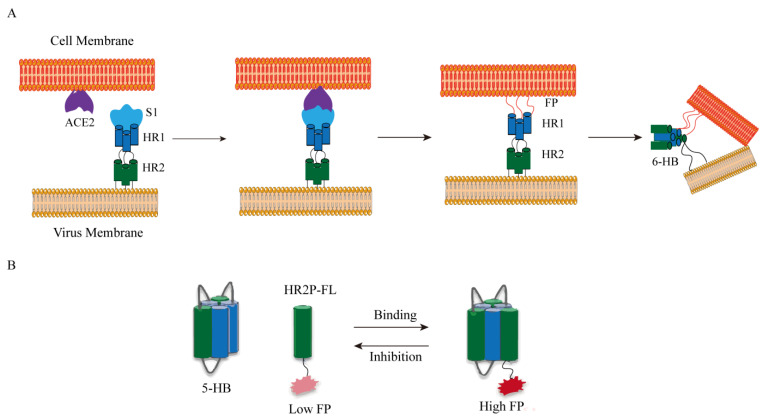
(**A**) SARS-CoV-2 membrane fusion mechanism. Abbreviations: FP, fusion peptide; HR, heptad repeat. (**B**) Fluorescence polarization (FP) determination. The FP value of HR 2 P-FL increased after the combination of 5-HB and HR 2 P-FL. Inhibitors could destroy the binding between 5-HB and HR2P-FL, decreasing the FP value.

The basic concept of fluorescence polarization (FP) is to connect the fluorophore to the ligand. The fluorophore-ligand conjugate has a low molecular weight and rotates freely in the solution at a high speed to emit a lower FP signal. After the addition of the receptor (usually a protein with a higher molecular weight), the binding between the receptor and the ligand noncovalently results in the restriction of the rotation so that a high FP signal is emitted (Figure 8). Xinjian Yin’s research team developed a high-throughput screening method for SARS-CoV-2 fusion inhibitors based on fluorescence polarization. As shown in Figure 8, the RBD of S1 is responsible for binding with ACE2, triggering a conformational change in the S2 subunit. Previously buried hydrophobic fusion peptides are then exposed and inserted into the host cell membrane. Subsequently, two heptad repeat sequences (HR 1 and HR 2) form a favorable six-helix bundle (6-HB) post-fusion structure, bringing the virus and cell membrane together. Inspired by this, they constructed a plasmid with a short linker and ligated three HR 1 and two HR 2 of SARSCoV-2 to obtain a 5-HB structure. This structure has a high binding affinity with fluorescein-5-maleimide-labeled HR 2 peptide (HR2P-FL). After HR2P-FL binds to 5-HB, the rotation is limited, thereby enhancing the fluorescence polarization signal. The binding of 5-HB to HR2P-FL is disrupted by the presence of fusion inhibitors, resulting in a decrease in FP value, thereby rapidly identifying spike inhibitors that bind to ACE2 [52].

### 3.2. Fluorescence-Based Drug Screening Method Targeting 3CLpro and PLpro

The 3CLpro, also known as the main protease (Mpro) [53,54], is a cysteine protease composed of 306 amino acids and consists of three domains (domains I to III). In SARS-CoV-2, the 3CLpro contains a Cys-His catalytic dimer (Cys 145 and His 41) between domains I and II. This catalytic dimer has the unique ability to specifically recognize 11 cleavage sites of nsp4-16, resulting in the release of coronavirus nsp. The nsp4–nsp16, which is released through hydrolysis and cleavage by 3CLpro, serves as a vector for viral genome replication and transcription. Moreover, it plays a crucial role in protein cleavage and modification, as well as post-translational nucleic acid synthesis [55]. PLpro (papain-like protease) is a versatile protein that exhibits dual functionality as both a protease and a phosphatase. It possesses the capability to modulate the immune response, counteract interferon (IFN) molecules, and actively engage in viral replication [56]. The PLpro enzyme cleaves the viral poly-proteins, resulting in the liberation of nsp1, nsp2, and nsp3. This step is crucial for the replication of the virus [57,58,59].

Therefore, it is promising to develop anti-SARS-CoV-2 drugs targeting 3CLpro and PLpro. At present, many high-throughput screening methods for 3CLpro and/or PLpro inhibitors based on fluorescence have been developed.

**Figure 9:**
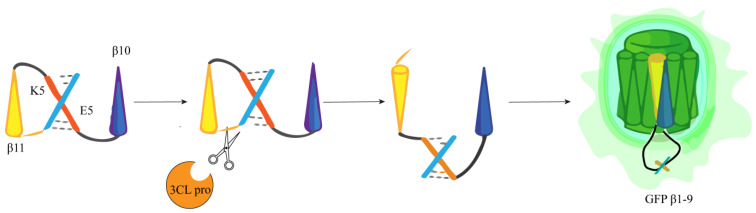
Diagram of the FlipGFP protease reporter.

The research team of Heather M. Froggatt developed a fluorescence high-throughput screening method in response to the main proteases of SARS-CoV-2. They started with the FlipGFP protein, FlipGFP, by expressing green fluorescent protein (GFP) 10 and 11β chains (GFP β10–11), separated from the rest of the GFPβ barrel (GFPβ1–9) and in an incompatible inactive conformation. The adaptor containing the cleavage site maintains the two GFPβ chains (β10–11) in an inactive parallel conformation and cuts the adaptor containing the cleavage site when an appropriate main protease cleavage enzyme is present. This cleavage allows GFPβ10–11 to reorientate to form an antiparallel conformation and is able to adapt to GFPβ1–9, so binding occurs, inducing fluorescence to exceed the background by 100 times (Figure 9), thereby determining protease activity based on changes in fluorescence signal [60]. The reporter has good compatibility with many CoV 3CLpro proteins and supports rapid testing of inhibitors against various coronavirus 3CLpro proteins without the need to synthesize protease substrates or purify viral proteins. Since this assay is performed in living cells, cell viability can also be measured while screening protease inhibitors [61].

**Figure 10:**
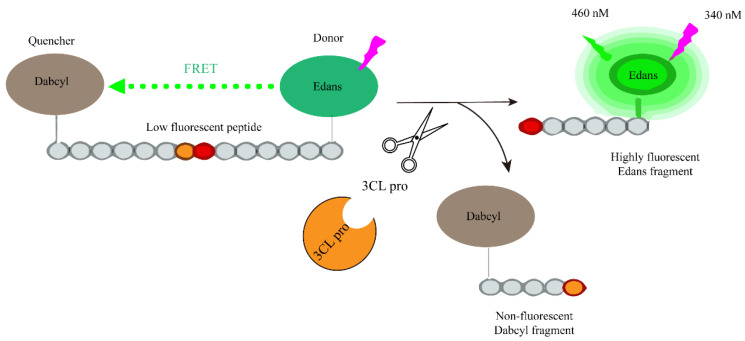
Schematic representation of the fluorogenic assay for the enzymatic activity of the SARS-CoV-2 protease.

Wei Zhu et al. developed a fluorescence-based high-throughput screening method to determine the activity of SARS-CoV-2 3CLpro using FRET (Fluorescence Resonance Energy Transfer). As depicted in Figure 10, a peptide substrate was constructed with an energy donor, Edans, connected at the C-terminus, and an energy acceptor, Dabcyl, connected at the N-terminus. In this configuration, the system exhibited low fluorescence due to the quenching of Edans by Dabcyl. However, in the presence of 3CLpro, it cleaves the peptide substrate, separating the C-terminal and N-terminal, disrupting the energy transfer between the quenching group (Dabcyl) and the fluorophore (Edans). Consequently, this disruption leads to an increase in the fluorescence signal. The magnitude of the fluorescence increase is directly proportional to the activity of 3CLpro [62]. Therefore, in the presence of potential 3CLpro inhibitors, the increase in fluorescence signal will either disappear or weaken. This enables the high-throughput screening of active compounds.

**Figure 11:**
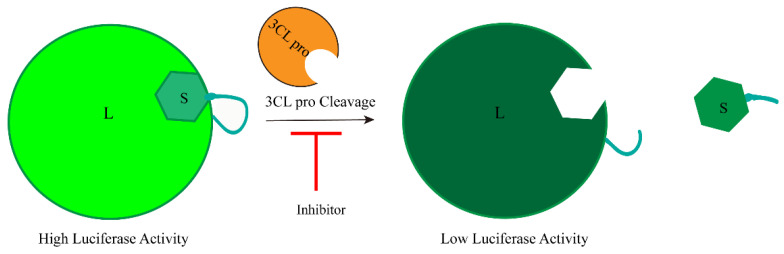
Development of luciferase complementary reporters based on cell-based inhibition of SARS-CoV-2 3CLpro activity.

Jonathan M.O. Rawson et al. designed a screening strategy for 3CLpro inhibitors using complementary luciferase technology. They used a complementary luciferase called Nano-BiT, which consists of a large BiT (L) and a small BiT (S) luciferase complementary reporter. Additionally, they introduced a 3CLpro cleavage site adaptor to the two components. When the L and S fragments are connected through the adaptor, they re-form the NanoBiT complex, resulting in increased luciferase activity. Conversely, cleavage by 3CLpro disrupts the complementarity between L and S, leading to decreased luciferase activity (Figure 11) [63]. Hence, the presence of the 3CLpro inhibitor impedes its cleavage, thereby reversing or influencing the luciferase inactivation and partially restoring the fluorescence signal.

**Figure 12:**
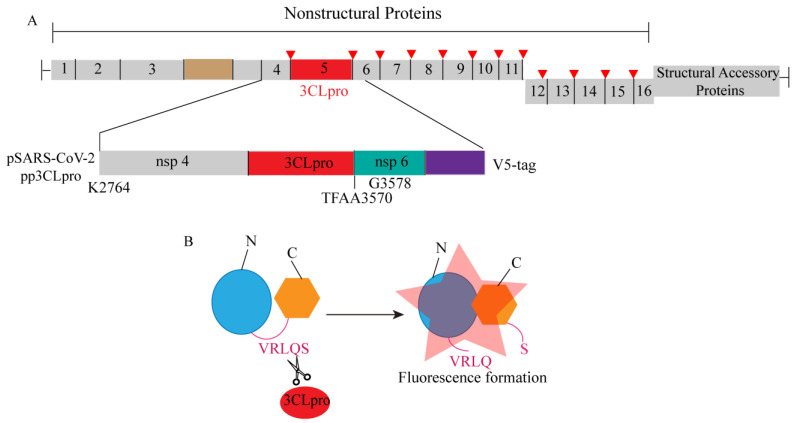
A schematic diagram illustrating the development of an enzyme-based biosensor for evaluating the activity of SARS-CoV-2 3CLprotease (3CLpro). (**A**) The schematic diagram depicts the region of SARS-CoV-2 nonstructural proteins 4 and 5, as well as the amino-terminal region of nsp6, which were cloned into the pcDNA3.1 expression vector with an in-frame V5 epitope tag. (**B**) The schematic diagram of the pGlo-VRLQS biosensor activated after cleavage by 3CLpro.

Amornrat O’Brien’s research team previously developed a luminescence-based biosensor for evaluating the activity of MERS-CoV 3CLpro. This biosensor, based on the circular arrangement of firefly luciferase, maintains its inactivity through a flexible linker. In order to assess the effectiveness of their previously established biosensor (pGlo-VRLQS) for SARS-CoV-2 3CLpro, the insertion of the 3CLpro target site (VRLQS) in the flexible linker region allows protease cleavage, leading to conformational changes in the protein and resulting in bioluminescence, to construct a plasmid called pp 3CLpro, which expresses the amino-terminal segment of nsp4, nsp5, and nsp6 (Figure 12A). Previous studies have shown that pp 3CLpro allows autocatalytic processing and release of 3CLpro, and then the released 3CLpro can cleave the conserved sequence (VRLQ/S) in the biosensor, leading to its activation and generation of fluorescence signals (Figure 12B). Therefore, the increased amount of pp 3CLpro plasmid DNA was transfected into cells containing biosensors, resulting in a dose-dependent increase in luciferase activity [64]. Following the optimization of screening conditions, the established biosensor (pGlo-VRLQS) can be effectively utilized for high-throughput screening of inhibitors targeting the SARS-CoV-2 3CLpro enzyme. Additionally, the research team observed that the activity of the SARS-CoV-2 3CLpro enzyme closely resembles that of the Middle East respiratory syndrome coronavirus (MERS-CoV) 3CLpro enzyme, possibly attributed to the substantial similarity in their gene sequences [65,66].

**Figure 13:**
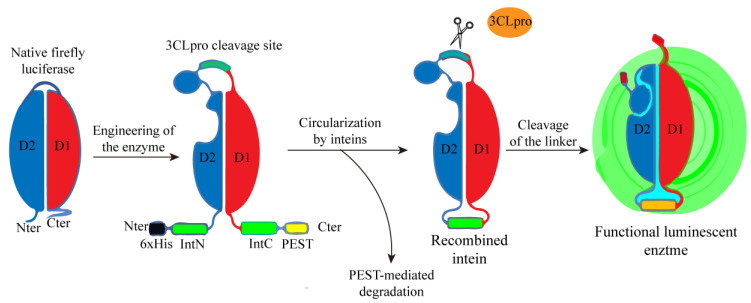
Schematic design of a luminescent biosensor for detecting the activity of 3CLpro.

Cyrille Mathieu’s research team also developed a biosensor based on bioluminescence to detect the proteolytic activity of SARS-CoV-2 3CLpro. They utilized the 3CLpro cleavage site-linked firefly luciferase constructs with circularly arranged N-terminal fragments (4-233 D1) and C-terminal fragments (234-544 D2), and stabilized the biosensor by catalyzing the cyclization of the head-to-tail-linked N and C-terminal intein domains IntN and IntC. At the same time, in order to forcibly eliminate proteins that fail to be cyclized and may produce background signals, PEST degradation sequences were added to the C-terminus of the construct to induce the degradation of uncyclized proteins. This circular arrangement locks the luciferase in an open conformation and prevents the luciferase from processing to inactivate it. When the adaptor is cut by 3CLpro, the binding domain is relaxed, allowing the conformation to change into a closed conformation and fold into a functional luciferase, thereby restoring luciferase activity (Figure 13) [67]. In the presence of 3CLpro inhibitors, the above changes are affected, and compounds or drugs with 3CLpro inhibitory activity can be screened by measuring the generated luminescent signal.

**Figure 14:**
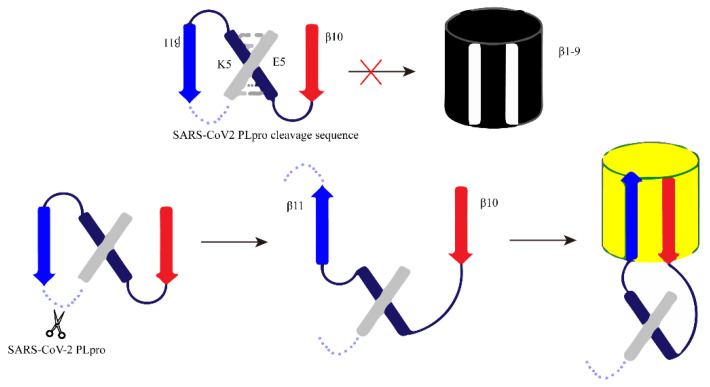
The schematic diagram of the design principle of FlipGFP detection based on cells. Red cross: the configuration is not suitable, and it cannot bind to produce fluorescence.

Consistent with the method used by Heather M. Froggatt’s research team, Chunlong Ma et al. separated the β chain 10–11 of GFP protein from the rest of the GFP β barrel (β chain 1–9). The 10th and 11th β chains are connected to the heterodimerization coiled coil E5/K5 through the PLpro cleavage site. In the absence of PLpro, the 10th and 11th β chains are restricted, the parallel conformation cannot bind to the GFP β barrel 1–9, and there is no luciferase activity. When the cleavage site is cleaved by PLpro, the 11th β chain then flips its direction in reverse parallel to the 10th β chain and associates with GFPβ barrel 1−9 to restore luciferase activity, resulting in green fluorescence signal recovery (Figure 14) [68].

**Figure 15:**
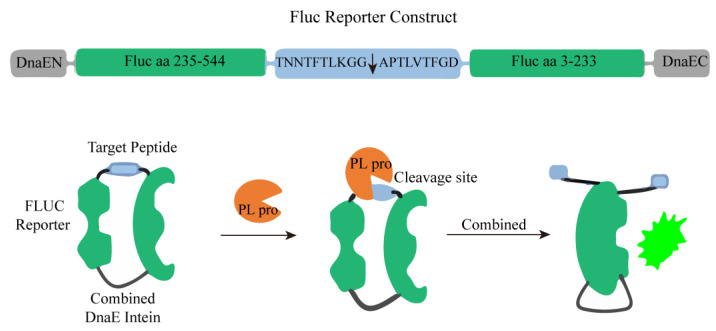
A schematic diagram of PLpro activity detection based on firefly luciferase (FLuc) reporter was constructed.

Emery Smith’s team found drugs that inhibit PLpro by using transiently transfected cells with PLpro and an endonuclease-based reporter gene. As shown in Figure 15, the C-terminal (FLuc aa 3-233) and N-terminal (FLuc aa 235-544) parts of the firefly luciferase (FLuc) gene are separated by a target peptide containing a cleavage sequence at the nsp2 and nsp3 junctions, which are connected by DnaE to form a ring. After the addition of PLpro, the N-and C-terminal DnaE inteins are cleaved, the FLuc domain is dimerized and has catalytic activity, and the luminescent signal can be detected [69]. In the presence of potential PLpro inhibitors in the screening system, the increase in the luminescent signal value may be weakened or disappear.

**Figure 16:**
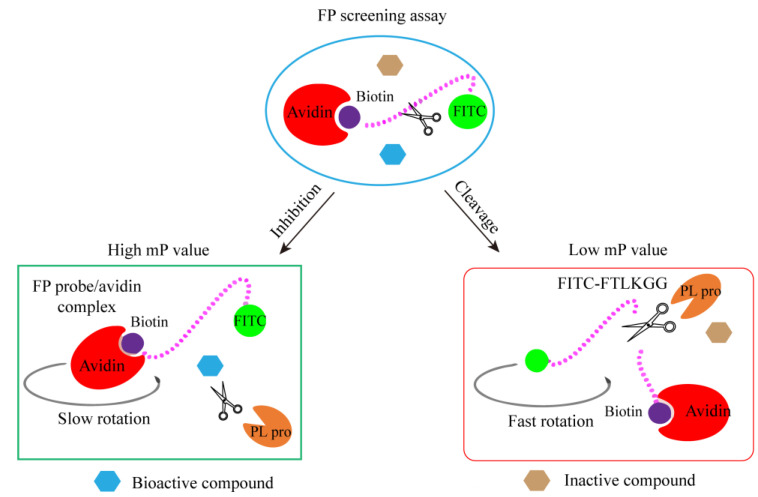
The schematic diagram of sandwich-like FP screening method based on fluorescence.

Haohao Yan et al. developed a straightforward and robust sandwich-like fluorescence polarization (β-FP) screening assay for the identification of PLpro inhibitors (Figure 16). In this fluorescent protein screening experiment, synthetic peptides with similar sequences in the FRET experiment were coupled with fluorescein isocyanate (FITC) fluorophore and biotin to produce a fluorescent protein probe FITC-FTLKGGAPTKVTK-biotin conjugate. Subsequently, the FP probe was incubated with PLpro (scissors), and avidin protein (red crescent) was added. The complete FP probe conjugate was combined with avidin protein to form a large binding complex, resulting in a high mP value due to slow rotation. PLpro cuts the FP probe to release the fluorescent small molecule FITC-FTLKGG, which has a low mP value due to rapid rotation. When there is a bioactive compound (blue hexagon) that inhibits the activity of PLpro, the cleavage of the probe by PLpro is inhibited. Consequently, the structure of the large binding complex is preserved, maintaining a high mP value. The presence of inactive compounds (brown hexagon) does not interfere with the cleavage of PLpro, resulting in a low mp value. [70]. Therefore, by monitoring the change in the mP value, candidate compounds in the extensive compound library can be promptly identified.

**Figure 17:**
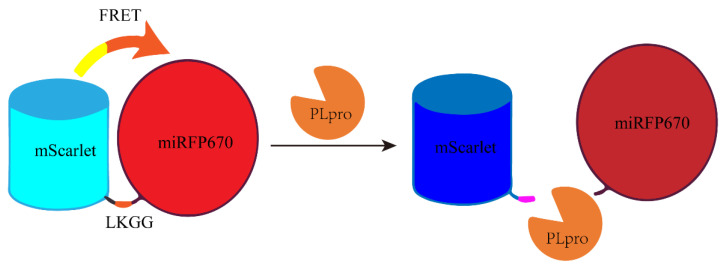
A schematic diagram of a fluorescent protein sensor composed of FRET donor mScarlet and receptor miRFP670 connected by LKGG.

Elena L et al. designed a red-shifted gene-encoded sensor using FRET (Förster resonance energy transfer) with green fluorescent protein (GFP) as the donor and biliverdin binding near-infrared fluorescent protein (BIR) as the acceptor. As depicted in Figure 17, the PLpro sensor consists of a fluorescent protein mScarlet and miRFP670 connected by a linker (LKGG recognition site of PLpro), which allows FRET and subsequent fluorescence quenching. When PLpro is potentially present in the system, it cleaves the linker, disrupting FRET and causing the fluorescent group and quenching group to move apart. Consequently, the fluorescent group emits fluorescence again, resulting in an increase in the fluorescence intensity of mScarlet [71]. The activity of PLpro was measured by the increase in the fluorescence signal value. On the contrary, when its activity was inhibited, it could also be reflected in the decrease of the fluorescence signal value.

### 3.3. Fluorescence-Based Drug Screening Method Targeting RdRp

RNA-dependent RNA polymerase (RDRP) is an important enzyme that facilitates RNA synthesis by catalyzing the formation of RNA template-dependent phosphodiester bonds. The replication of SARS-CoV-2 genome and its gene transcription are mainly controlled by viral RNA-dependent RNA polymerase (RdRp), and there is no protein in the host that can perform the same function. Therefore, RdRp is considered to be a very promising target in drug development [24,72,73,74]. A rapid, convenient and effective screening method for RdRp inhibitors will greatly accelerate the development of RdRp-targeted drugs.

**Figure 18:**
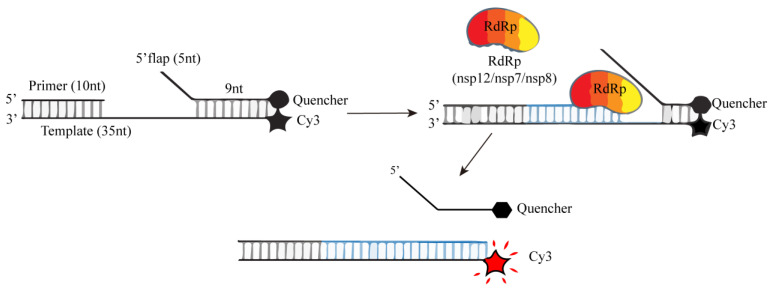
A schematic diagram of RdRp chain displacement determination based on FRET.

In order to evaluate the activity of RdRp, Agustina P. Bertolin’s research team developed a FRET-based chain displacement detection method (Figure 18). They hybridized a 35-nucleotide RNA template, which incorporated a Cy3 fluorophore at the 5′ end, and a quenching group on the complementary 14 nt quenching chain at the 5′ end. This design ensured that the RNA substrate did not exhibit any Cy3 fluorescence signal. Additionally, they introduced a 10 nt primer chain at the corresponding position on the 3′ end of the template, generating a primer RNA substrate. The active RdRp will elongate the primer chain and thus replace the downstream quencher chain. The loss of the quencher chain will terminate the FRET effect between the fluorophore and the quenching group, thereby generating a fluorescence signal because of the final double strand without the quenching group. Thus, preventing the reannealing of the quencher chain, maintains the generated fluorescence signal [75]. The increase in fluorescence signal value is positively correlated with the activity of RdRp. When there is an inhibitor of RdRp, the increase in fluorescence signal value will weaken or disappear, which is conducive to the rapid screening of RdRp inhibitors.

**Figure 19:**
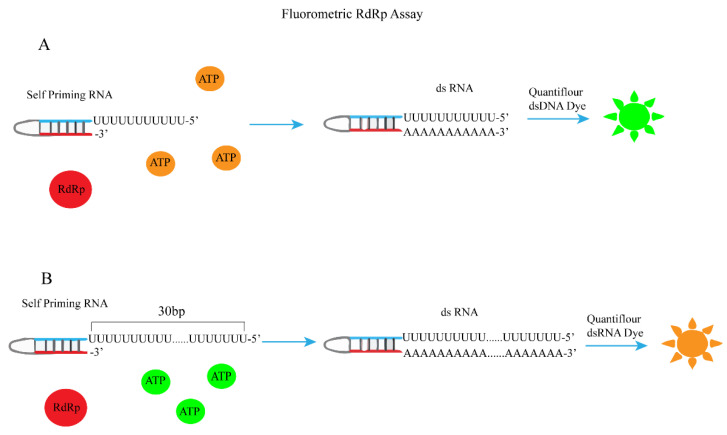
A schematic diagram for the determination of RDRP activity using self-initiating RNA, Quan-tiflour dsDNA, or Quan-tiflour dsRNA fluorophores. (**A**) Schematic of fluorometric RdRp assay with self-priming RNA and Quantiflour dsDNA fluorophore. (**B**) Schematic of fluorometric RdRp assay with self-priming RNA and Quantiflour dsRNA fluorophore.

The most important function of RdRp is to catalyze the RNA template synthesis of RNA [76,77]. Therefore, fluorophores that can distinguish single-stranded RNA and double-stranded RNA can be used to evaluate RdRp activity. Based on this principle, Kocabas ·Raife D et al. used RdRp to catalyze self-initiated RNA synthesis of double strands and then measured RdRp activity by dsRNA quantification (Figure 19A) [78]. First, they evaluated a series of commonly used fluorophores (Quantiflour RNA, Quantiflour dsDNA, and picoGreen systems) and found that the Quantiflour dsDNA system has a consistent dsRNA/ssRNA ratio and is suitable for measuring the formation of dsRNA from ssRNA after RdRp catalysis, which can provide an ideal fluorophore for quantifying dsRNA formed during RdRp reaction. Next, the following complementary ssRNAs are used for dsRNA generation:

Positive ssRNA: 5′-UUUUUUUUUUUAACAGGUUCUA-3′

Antisense ssRNA: 5′-UAGAACCUGUUAAAAAAAAAAA-3′

Finally, the addition of RdRp catalyzes the synthesis of double-stranded RNA, and dsRNA is quantified by Quantiflour dsDNA fluorescence system. The change in fluorescence signal value can be used to determine the activity of RdRp. Thus, the establishment of a fluorescence screening method for RdRp inhibitors was achieved. Xiaoming Bai et al. also used a similar principle but a different fluorescent dye and a different self-priming sequence to establish the corresponding RdRp fluorescence high-throughput screening method (Figure 19B) [79].

**Figure 20:**
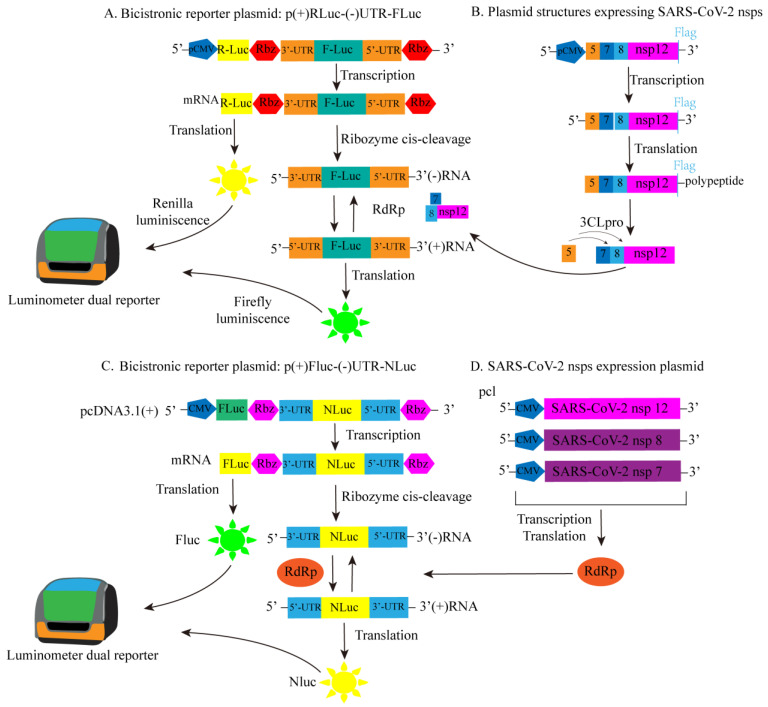
The constructed SARS-CoV-2 RdRp bicistron RdRp reporter substructure diagram. (**A**) Plasmid for dual luciferase reporter: schematic diagram of p(+)RLuc-(−)UTR-NLuc. (**B**) Express the plasmid structure diagram of SARS-CoV-2 RdRp (nsp12), accessory proteins (nsp7 and nsp8), and viral 3CLpro protease (nsp5). (**C**) Plasmid for dual luciferase reporter: schematic diagram of p(+)FLuc-(−)UTR-NLuc. (**D**) Illustration of SARS-CoV-2 nsps expression plasmid.

A cell-based luciferase reporter system for SARS-CoV-2 RdRp was developed based on a similar principle to the previously reported cell-based assays for HCV RdRp and MERS-CoV RdRp activity [80,81]. To transiently express SARS-CoV-2 RdRp in mammalian cells, Timsy Uppal’s group constructed Flag-labeled vectors for nsp5, nsp7, nsp8, and nsp12. The constructed Flag-labeled vectors expressed RdRp along with other auxiliary proteins, namely nsp7, nsp8 (an auxiliary protein with RdRp activity), and 3CLpro (nsp5 with autoproteolytic activity), as shown in Figure 20B. Subsequently, a bicistronic reporter gene for SARS-CoV-2 RdRp was constructed. As depicted in Figure 20A, the firefly luciferase (FLuc) and renilla luciferase (RLuc) genes are arranged in a reverse orientation, flanked by the 5′-UTR and 3′-UTR of SARS-CoV-2, along with the ribozyme self-cleavage sequence from hepatitis D virus (HDV). The host RNA polymerase Pol II is responsible for transcribing the full-length (+) RLuc- (−) UTR-FLuc RNA. These bicistronic RNA transcripts are then subjected to self-cleavage by ribozymes, leading to the expression of FLuc RNA in the opposite direction. Consequently, the RdRp polymerase is able to transcribe the sense FLuc RNA for protein synthesis. Therefore, the measured FLuc fluorescence signal intensity is directly proportional to the intracellular RdRp activity. Additionally, in the presence of RdRp activity, the synthesis of (+) Fluc RNA is amplified, while the levels of RLuc RNA (used as an internal control for transcription and translation) remain unaffected. Any compounds that inhibit RdRp activity will cause a decrease in FLuc levels, serving as an indicator of their inhibitory effects [82]. So, they established a cell-based fluorescence high-throughput screening method for anti-SARS-CoV-2 antiviral drugs.

Jung Sun Min’s research team utilized a similar approach to the screening method mentioned above, working with reporter gene plasmids created through modifications of the previously published dicistronic MERS-CoV RdRp reporter gene construct (Figure 20D). The key distinction lies in their choice of using NLuc fluorescence signal value to indicate the activity of SARS-CoV-2 RdRp, while FLuc serves as an internal control (Figure 20C) [83].

### 3.4. Fluorescence-Based Drug Screening Method Targeting Helicase

One of the key participants in the replication of the SARS-CoV-2 genome is the virus-encoded RNA helicase Nsp13 (Helicase). Helicase combines with Nsp12 (RdRp), continuous synthesis factors (Nsp7, Nsp8) and proofreading exonuclease (Nsp14), which is responsible for the replication and transcription of the viral genome [84,85]. SARS-CoV-2 helicase is a 5′→3′ translocated helicase, which is powered by deoxyribonucleotide triphosphate. It acts on deoxyribonucleic acid substrates, catalyzes the unwinding of double-stranded RNA and then replicates in large quantities, playing a key role in viral genome replication [86,87,88].

**Figure 21:**
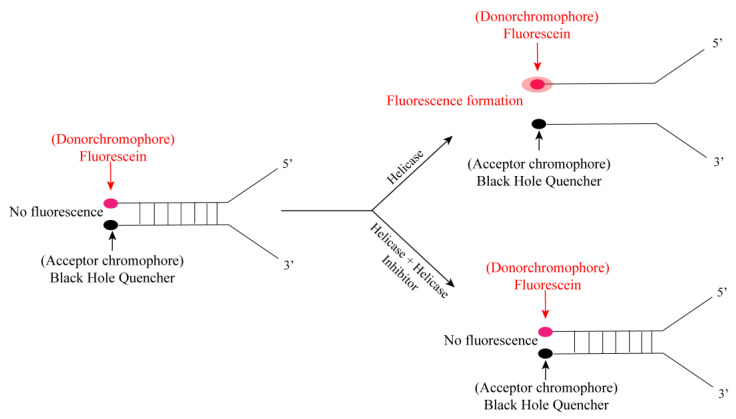
Schematic diagram of fluorescence determination of helicase activity.

In 2012, Adeyemi O. Adedeji et al. proposed the principle of fluorescence screening method for the helicase of severe acute respiratory syndrome (SARS) caused by SARS-CoV. As shown in Figure 21, there are two chromophores (fluorescein and black hole quencher) at the end of the complementary strand of the dsDNA substrate (fork substrate). In the presence of active helicase, the two complementary strands are separated, and the fluorophore and the quencher are far away from each other, allowing fluorescence to occur. In the presence of a potential helicase inhibitor, the two complementary strands will not be separated; therefore, fluorescence will not be detected [89].

**Figure 22:**
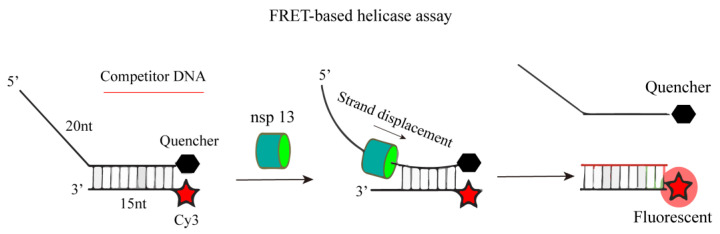
A schematic diagram for the determination of nsp13 helicase activity based on FRET.

Inspired by the determination method of SARS-CoV helicase activity, researchers have developed a method for the determination of SARS-CoV-2 helicase. This principle was applied to the screening of SARS-CoV-2 helicase in 2021. Jingkun Zeng’s research team developed a FRET-based helicase screening method. As shown in Figure 22, they heat-treated the fluorophore-labeled oligonucleotide (Cy3 chain) with the oligonucleotide containing the quencher (BHQ-2 chain) to produce a nucleic acid substrate with 50 nt. Nsp13 (Helicase) uncoils, and the competing oligonucleotides capture the free Cy3 chain, preventing the substrate from reannealing, and the FRET effect between the fluorophore and the quencher disappears, resulting in a fluorescence signal [90]. When there is a potential helicase inhibitor, the double strand cannot be unlocked, and the existence of the FRET effect makes the nucleic acid substrate unable to produce fluorescence.

At present, many fluorescent screening methods have been developed for Spike, 3CLpro, PLpro, RdRp, and Helicase, and their feasibility has been verified by known inhibitors; many of these methods have also identified many new viral target inhibitors, as shown in Table 3. This indicates that the establishment of efficient and rapid fluorescent screening methods for five viral targets is crucial for finding new anti-SARS-CoV-2 drugs.

## 4. Conclusions

In 2019, an outbreak of novel coronavirus pneumonia, termed COVID-19, emerged in Wuhan, Hubei Province, China, attributed to the SARS-CoV-2 virus. Owing to its pronounced contagiousness, the virus rapidly disseminated worldwide, precipitating a global health crisis characterized by extensive infections and pandemics. The profound infectivity, severe clinical manifestations, and enduring repercussions of this virus have inflicted substantial detriment upon global public health infrastructures, human well-being, and economic stability. Consequently, intensive research endeavors have been directed toward the identification and development of anti-SARS-CoV-2 therapeutics, owing to the recognition of five pivotal viral protein targets—Spike, 3CLpro, PLpro, RdRp, and Helicase—that are integral to the virus’s infection and replication mechanisms. The successful formulation of efficacious anti-SARS-CoV-2 agents targeting these proteins represents a significant advancement in the field. This necessitates the establishment of high-throughput screening protocols focused on these five viral proteins. Such methodologies are instrumental in the expedited evaluation of existing drugs, clinical candidates, natural compounds, and chemical libraries to discern potential inhibitory agents, thereby serving as foundational leads for the development of innovative anti-SARS-CoV-2 medications. To this end, the present review delineates fluorescence-based screening techniques for Spike, 3CLpro, PLpro, RdRp, and Helicase utilizing methodologies such as time-resolved fluorescence resonance energy transfer (TR-FRET), fluorescence resonance energy transfer (FRET), fluorescence enzyme complementation, bioluminescence sensors, among others. Furthermore, we elucidate the underlying design principles and fundamental concepts governing these screening approaches tailored for distinct viral proteins. It is our aspiration that this comprehensive overview will aid researchers in the judicious selection or refinement of screening methodologies tailored for combating SARS-CoV-2 effectively.

## Figures and Tables

**Table 1:** Novel coronavirus therapeutic drugs that have been approved worldwide.

Drug Name	Drug Type	Action Mechanism	Approved Areas
Amubarvimab/Romlusevimab	mAb	Spike protein inhibitors	China
Casirivimab/Imdevimab (Ronapreve)	mAb	Spike protein inhibitors	USA, EU, UK, Japan
Sotrovimab	mAb	Spike protein inhibitors	USA, EU, UK, Japan
Regdanvimab	mAb	Spike protein inhibitors	EU, South Korea
Bamlanivimab/Etesevimab	mAb	Spike protein inhibitors	USA
Tixagevimab/Cilgavimab (Evusheld)	mAb	Spike protein inhibitors	USA
Bebtelovimab	mAb	Spike protein inhibitors	USA
Tocilizumab	mAb	Cytokine antagonists	USA, Japan, EU
Remdesivir	Small molecule	RdRp inhibitors	USA, Japan, EU
Molnupiravir	Small molecule	RdRp inhibitors	USA, UK, Singapore
Favipiravir	Small molecule	RdRp inhibitors	Russia
Azvudine	Small molecule	RdRp inhibitors	China
VV116	Small molecule	RdRp inhibitors	Uzbekistan
Nirmatrelvir/Ritonavir	Small molecule	3CLpro inhibitors	China, EU, UK, USA, Japan
Ensitrelvir	Small molecule	3CLpro inhibitors	Japan
Baricitinib	Small molecule	JAK1 and JAK2 inhibitors	USA, Japan
Proxalutamide	Small molecule	Androgen antagonist	Paraguay
2-Deoxy-D-glucose	Small molecule	Glucose metabolism inhibitors	India
Dexamethasone	Small molecule	Glucocorticoids	UK

**Table 2:** Several key viral proteins and enzymes in the life cycle of SARS-CoV-2 and their main functions.

Virus Protein or Enzyme	The Main Functions in the Life Cycle of SARS-CoV-2
Spike	Mediates the binding of virus to host receptor
3CLpro/PLpro	Cleaves multiproteins to produce nonstructural proteins (NSPs) such as RdRp and Helicase.
RdRp	It is involved in the transcription and synthesis of negative-strand subgenomic RNA, the synthesis of mRNA related to different structural proteins, and the replication of viral genomic RNA.
Helicase	Unwind the positive and negative strands of viral RNA for viral RNA replication.

**Table 3:** Several key viral proteins and enzymes in the life cycle of SARS-CoV-2 and the fluorescent screening methods established around them and the compounds and drugs screened or verified.

Virus Protein or Enzyme	The Established Fluorescence Screening Method	Potential Compounds Were Identified by Corresponding Fluorescence Screening Methods or Compounds with Known Inhibitory Activity Were Verified
Spike	RBD-ACE 2 binding assay based on TR-FRET	Cangrelor, Elaidic Acid, Fenbendazole, Enalaprill, Maleate, Corilagin
	ACE2 biosensor based on fluorescence resonance energy transfer (FRET)	HCQ, MLN-4760
	Fluorescence polarization method for screening SARS-CoV-2 fusion inhibitor	Salvianolic acid A, Salvianolic acid B, Salvianolic acid C, Rosmarinic acid, Lithospermic acid, Caffeic acid
	Cell-based entry detection of SARSCoV-2 using viral pseudotype and internalized luciferase technology	Dichlorophen, Calpeptin, Aloxistatin, CAA-0225, Brigatinib, VBY-825
	Biosensor based on nano-luciferase complementation: SARS-CoV-2 S1 NanoBiT	Theaflavin, Baicalin, Hesperidin, Sculellarin, Heparin, VHH72
	Fluorescent reporters capable of identifying compounds that inhibit SARS-CoV-2	Homoharringtonine, Triptolide, Oleandrin, Bufalin, Anisomycin, Harmine, Doxorubicin, Cinobufagin, Resibufogenin, Periplocin, Triptonide, Bufotaline, Bruceine A, Bruceine D, Eurycomanone etc.
	Luciferase-based quantification of membrane fusion induced by SARS-CoV-2 S protein	Nafamostat, K874A
3CL pro	Fluorescence reporter gene assay based on green fluorescent protein (GFP) derivative	GC 376
	Cell luciferase complementary screening test	Boceprevir, Z-FA-FMK, Calpain Inhibitor XII, GRL-0496, GC376
	Firefly luciferase bioluminescent sensor screening method	Setanaxib, Tretinoin, Nepicastat, Balicatib, Vidofludimus, KNK437, Clarithromycin
	SARS-CoV-2 3CLpro polyclonal antiserum and biosensor detection	GRL-0496
	Detection of SARS-CoV-2 3CL pro activity by FRET-based fluorescence method	GC376, Walrycin B, Hydroxocobalamin, Z-DEVD-FMK, Suramin sodium, LLL-12, Z-FA-FMK, Anacardic Acid, Adomeglivant, Eltrombopag olamine, GSK-3965, GW5074, Hexachlorophene, MK-886, AMG-837, MG-149
PLpro	Luciferase complementary assay	Bleomycin, Ripasudil, Rho-kinase-IN-1, and R-547
	FlipGFP reporter gene fluorescence screening method	GRL 0617, Jun 9 -72-2, Jun 9 -75-4
	Fluorescence polarization screening method	Anacardic acid, GRL0617
	FRET detection method based on red fluorescent protein mScarlet as donor and biliverdin binding near-infrared fluorescent protein miRFP 670 as receptor.	-
RdRp	Screening method of SARS-CoV-2 RdRp chain replacement based on FRET	GSK-650394, C646, BH3I-1, MDK-83190, Cefsulodin, Suramin
	Fluorescence RdRp detection method based on self-priming RNA	Remdesivir, Triphosphate remdesivir, C646, BH3I1
Helicase	FRET-based SARS-CoV-2 nsp13 helicase assay	FPA-124, Suramin, NF023, Navitoclax, Linoleic acid, SSYA10-001, Myricetin

## Data Availability

Not applicable.
